# Excessive Smartphone Use Is Associated With Health Problems in Adolescents and Young Adults

**DOI:** 10.3389/fpsyt.2021.669042

**Published:** 2021-05-28

**Authors:** Yehuda Wacks, Aviv M. Weinstein

**Affiliations:** Department of Behavioral Sciences, Ariel University, Ariel, Israel

**Keywords:** internet addiction, smartphone addiction, problematic smartphone use, internet use disorder, excessive smartphone use

## Abstract

**Background and Aims:** This present paper will review the existing evidence on the effects of excessive smartphone use on physical and mental health.

**Results:** Comorbidity with depression, anxiety, OCD, ADHD and alcohol use disorder. Excessive smartphone use is associated with difficulties in cognitive-emotion regulation, impulsivity, impaired cognitive function, addiction to social networking, shyness and low self-esteem. Medical problems include sleep problems, reduced physical fitness, unhealthy eating habits, pain and migraines, reduced cognitive control and changes in the brain's gray matter volume.

**In Conclusion:** Excessive smartphone use is associated with psychiatric, cognitive, emotional, medical and brain changes that should be considered by health and education professionals.

## Introduction

### Excessive Smartphone Use in Young Adults

The effects of excessive use of computer screens and smartphones are raising serious concerns among health and educational authorities due to negative effects of such use in children and adolescents. Recent reviews have argued that the evidence supporting excessive smartphone use as an addictive behavior is scarce. In particular, Billieux (1) have argued that there is insufficient evidence for behavioral and neurobiological similarities between excessive smartphone use other types of addictive behaviors. Panova and Carbonell (2) also argued that there is insufficient evidence to support for the diagnosis of smartphone addiction and finally Montag et al. (3) have argued that excessive smartphone use is a form of Internet Use Disorder. The smartphones are being used for various purposes such as gaming, Social Network Services (SNS), watching video clips (YouTube). Therefore, excessive use of smartphones may have difference characteristics according to the type of smartphone use. This present paper will review the existing evidence on excessive smartphone use, and it will discuss its similarities with and differences from Internet addiction.

## Methods

A PubMed Central^®^ and Web of Science search engines have been used with the terms: “excessive smartphone use” and “smartphone addiction” until February 2021 that resulted in 84 research studies in English language.

### Predictors of Excessive Smartphone-Use

The main factors predicting excessive smartphone use were being female, preoccupation, conflict, and use for ubiquitous trait whereas the protective factor was use for learning (4). Excessive use of smartphones was correlated with impairment in the function of the family and relationship with friends, impulsiveness, and low self-esteem in South Korean adolescents (5). Finally, smartphone gaming was associated with excessive smartphone use among adolescents (6).

### Sensation Seeking and Boredom

Turgeman et al. (7) have reported an interaction between high sensation seeking and abstinence whereby abstinence for 1.5 h increased excessive smartphone use ratings in high sensation seeking students. This may be explained by boredom, avoidance of uncomfortable situations and the need for entertainment (8–12). Lepp et al. (13) have found an association between excessive smartphone use and living sedentary life or being an “active couch potato. “Ben-Yehuda et al. (14) have investigated the effects of involvement and of interest in three conditions: state of boredom, passive activity and active activity in counter-balanced order in University students. Excessive smartphone use was not influenced by any interest or involvement in the lecture, indicating a compulsive behavior. Finally, Li et al. (15) have demonstrated that individuals with an external locus of control had less control over their smartphone use and therefore could have more negative effects such as poor sleep quality, lower academic achievements, and lower ratings of well-being.

### Insecure Attachment, Poor Cognitive-Emotional Regulation and Communication Problems

Insecure attachment positively correlated with problematic smartphone use in students with unhealthy family function but not with mother-infant bonding or maternal mental health (16). Eichenberg et al. (17) showed an association between excessive smartphone use and an insecure attachment style in Problematic adolescent users. A following study reported high scores in maladaptive Cognitive-emotion regulation (CER) strategies such as self-blame, blaming of others ruminating and catastrophizing thoughts (18). Experiential avoidance (i.e., attempts to avoid thoughts, feelings, memories and physical sensations) has been associated with excessive smartphone use and social networks (19). Childhood emotional maltreatment correlated with problematic smartphone use in adolescents, and it was mediated by body image difficulties, depression, and social anxiety (20). Emotion regulation difficulties, unregulated eating, restrained eating, food addiction, and higher percent body fat were associated with excessive smartphone use among adolescents (21). Mahapatra (22) showed a strong association between both lack of self-regulation and loneliness on problematic smartphone use among adolescents that ultimately resulted in family, interpersonal conflicts, and poor academic performance. Among students, problematic smartphone users have shown high measures of worry and anger (23) whereas excessive reassurance seeking behavior mediated the association between rumination and problematic smartphone use (24). Poor communication skills were shown in Medical students who preferred to communicate emotions through texting rather than verbal communication (25) and they correlated with excessive smartphone use (26). Excessive use of the smartphone has negative impacts on people's lives by reducing face-to-face interactions, and increasing loneliness (27).

### Impaired Cognitive Function

Problems in inhibitory control mechanisms in excessive smartphone users were reported (28). They have reported that while performing on the Go/NoGo task excessive smartphone users showed a negative N2 event-related potentials (ERPs) component showing reduced inhibitory control. There is further evidence for impaired attention, reduced numerical processing capacity, increased impulsivity, hyperactivity and negative social concern in heavy smartphone users (29). Heavy smartphone users showed. Inattention problems correlated with Transcranial Magnetic Stimulation (TMS) evoked potentials in the right prefrontal cortex. Wegmann et al. (30) have found no correlations between problematic social networks use and executive function and inhibitory control measured by the Go/NoGo task. However, regression analyses showed that increased problematic social networks use is associated with higher impulsivity, especially if executive functions or specific inhibitory control were impaired.

### Social Media Use and Personality

Problematic social media use has been shown to be associated with “fear of missing out” (FOMO) (31, 32). FOMO mediated relations between both fear of negative and positive evaluation with both problematic and social smartphone use. Withdrawal and FOMO ratings were higher among participants with 72 h restricted access to smartphones compared with those without (33). There was a correlation between Social communication use and excessive use of smartphones. FOMO mediated the relationships between anxiety and depression with problematic smartphone use (24, 34). Excessive smartphone use has been associated with social comparisons on social networking sites and perceived stress (35). Personality factors such as conscientiousness, openness, emotional stability and neuroticism have been associated with problematic smartphone use (36, 37) whereas impulsivity, excessive reassurance seeking, but not extraversion related to problematic smartphone use in other studies (38, 39).

### Comorbidity With Anxiety, Depression OCD, ADHD and Alcohol Use Disorder

There are several studies on the comorbidity of excessive smartphone use and mental disorders and its association with sleep problems, reduced fitness and pain. Excessive smartphone use has been associated with depression, anxiety (40, 41) and social anxiety (7, 42–44) shyness and low self-esteem (5–12, 12–47) low psychological well-being (48) and low mental well-being (49). Excessive reassurance seeking correlated with problematic smartphone use severity, and its combination with rumination mediated the relationship between depression and anxiety severity with problematic smartphone use (50). Anxiety during the COVID-19 epidemic correlated with severity of problematic smartphone use, depression and generalized anxiety (51).

Early problematic smartphone use was found as a significant predictor of depression in a three-year longitudinal study from adolescence to emerging adulthood (52). Excessive mobile use was associated with high levels of depressive moods, with loneliness serving as a moderator of this mediation particularly in men (53). Depression and anxiety were significantly associated with both excessive smartphone use (54). Depressive mood and suicidal ideation were associated with social network smartphone use (55). Interestingly, the time spent in excessive smartphone use has predicted the level of stress in users who hardly used the smartphone for self-disclosure whereas those who engaged in disclosure of their emotions and problems online, this reduced their emotional problems (56). Problematic smartphone use has been associated with psychological distress and emotion dysregulation and emotion dysregulation was shown as a mediator in the relation between psychological distress and problematic smartphone use (57). Excessive smartphone use has been also associated with Obsessive Compulsive Disorder symptoms (58) and ADHD (59, 60).

History of alcoholism and father's education level explained 26% of the variance of problematic smartphone use (60). In addition, alcohol use disorder, impulsivity (Barratt scale and ADHD) and elevated occurrence of PTSD, anxiety, and depression were associated with excessive smartphone use (61). Finally, the relationship between PTSD severity and problematic smartphone use was mediated by negative urgency (a component of impulsivity) (62).

### Medical Complications- Sleep, Physical Fitness, Eyesight, Migraine and Pain

Excessive smartphone use was associated with reduced sleep time and sleep quality in adolescents (63). The association between media use in bed before sleep and depression was mediated by sleep disturbance (64, 65). Furthermore, there was an association between excessive screen time and problems in sleep onset (66), insufficient sleep (67), and insomnia (68). Long-term problematic mobile use predicted new incidences of sleep disturbances and mental distress, which was ameliorated by its discontinuation (69). Excessive mobile phone use correlated with disturbed sleep pattern and quality (70) Excessive smartphone use was associated with poorer sleep quality and higher perceived stress (71, 72), lowered physical activity, lower muscle mass and higher fat mass (73). Other medical conditions include acquired comitant esotropia (AACE) (74) increased ocular symptoms (75), headache complaints (76, 77) and headache duration and frequency in migraine patients (78). Young chronic neck pain patients with overuse of smartphones had higher Cervical Disc Degeneration (79). Finally, excessive smartphone users had higher median nerve Cross sectional areas (CSA's) in their dominant hands (80).

### Brain Imaging

A recent study has used diffusion MRI for assessment of white matter structural connectivity, and it has shown a positive association between activity in the right amygdala and excessive smartphone use in adolescents (81). Excessive smartphone users have shown impairment in cognitive control during emotional processing of angry faces and social interaction in fMRI (82). They also showed reduced functional connectivity in regions related to cognitive control of emotional stimuli including reward (83). Reduced Gray Matter Volume (GMV) was shown in problematic smartphone users and negative correlations between GMV in the right lateral Orbito Frontal Cortex (OFC) and measures of smartphone addiction (84). Lower activity in the right anterior cingulate cortex (ACC) and a negative correlation between individuals with excessive smartphone use and both ACC GMV and activity was reported (85). Furthermore, the strength of the resting state functional connectivity (rsFC) between several brain regions in fMRI positively correlated with smartphone time in bed (86). Finally, exposure to smartphone pictures in fMRI was associated with activation of brain regions associated with drug addiction and correlations of these regions with smartphone addiction scores were reported (87).

Supplementary Table 1 shows details of the studies reviewed in this paper.

## Discussion

There have been several reviews in recent years that have discussed the issue whether excessive smartphone use is considered a behavioral addiction (1, 2). In addition, studies have examined whether there are differences between excessive smartphone use and Internet use disorder (IUD). Montag et al. (3) have proposed that excessive smartphone use is essentially a type of IUD. In this sense, IUD should be divided into two types of use: a mobile use and a non-mobile use. They have suggested that there is a specific use of IUD of a particular content and a generalized IUD where several channels are overused. The rationale for this division is that motivation, cognitive and affective factors predispose individuals to prefer a specific application and type of device.

However, there is little empirical evidence in support of these assumptions (88, 89). Although there may be small differences between some mechanisms and risk factors underlying online behavioral addictions, such as pornography use, gaming disorder and social network use, the resemblance between them is very strong (90). In addition, there are few studies that have examined whether specific cognitive and motivational mechanisms could lead to a preference of a specific type of device. Nevertheless, recent studies show that excessive use of the screens including, computer screens and smartphones is associated with serious mental problems and cognitive impairments (91, 92). Therefore, we argue that research should focus on the negative consequences of excessive smartphone use rather than on whether it should be considered as a behavioral addiction.

Recent studies show that excessive smartphone use is associated with problems of mental health and impaired psychological well-being. There is consistent evidence for comorbidity between excessive smartphone use and other psychiatric disorders, such as depression, anxiety, OCD, and ADHD similar to Internet addiction (93). In addition, excessive smartphone use is related to loneliness, stress, and other negative emotions (56, 94).

In addition to these psychological consequences, the excessive use of smartphones can potentially lead to impairments of cognitive functions. Such excessive use is related to impairments of specific attention domains (such as focused attention and divided attention), low inhibitory control, impaired working memory, reduced numerical processing capacity, and changes in social cognition. Since cognition and emotion are often intertwined it is not surprising that a common cognitive-emotional mechanism related to loss of control would be associated with impulsiveness, impairment in communication and relationship with friends and family.

Recent studies have also shown an association between an excessive use of smartphones and abnormal activity of regions in the prefrontal cortex and in the networks that connect to these regions (29, 82). Novel findings show reduced lateral orbitofrontal gray matter, especially in social networking platforms overuse and that prolonged bedtime smartphone use has been associated with altered insula-centered functional connectivity. Gray matter volume reduction was observed also in the anterior cingulate similar to Internet and gaming disorder (95). Excessive smartphone use has also been associated with reduced cognitive control during the emotional processing in the brain.

The effects of excessive use of the media including TV, computer screens and smartphones is raising serious concerns among health and educational authorities due to deleterious effects of such use in children and adolescents. A recent study has shown an association between increased screen-based media use and lower microstructural integrity of brain white matter tracts that are associated with language and literacy skills in 5-year-old pre- school children, (96). Furthermore, a large study of 4,277 adolescents has shown a negative correlation between screen media activity and cortical thickness in fMRI implying premature aging of the brain (97). Finally, young adults and heavy media “multi-taskers” are more susceptible to interference from irrelevant environmental stimuli and from irrelevant representations in memory, and they performed worse on a task-switching ability (98). The findings so far that span from early childhood to adolescents, rapidly growing societal phenomena, emphasize the need to assess the effects of media screens on cognitive function and the brain in children, adolescents and young adults.

Excessive smartphone use shares underlying mechanisms with other addictive behaviors such as gambling disorder, in particular, reduced cognitive control and impaired activity in the prefrontal cortex which affects decision-making and emotional processing (99). Addictions in adolescents share the tendency to experience poor emotional regulation, impulsivity and impaired cognitive control and reduced ability to experience pleasure in everyday life (100).

The major limitations in studies of excessive smartphone use and Internet addiction are that they are mainly cross-sectional studies without baseline measures and rely on associations between structural and functional changes in the brain and subjective measures and no proof of a causal role in the development of the adolescent or adult brain. Finally, the review is non-systematic and it has excluded non-English language articles.

### Summary

The excessive use of the smartphone has been associated with impaired cognitive functions and mental health problems. There are unique findings on the association between using smartphones, need of constant stimulation, deficits in everyday cognitive functioning and brain changes which should send alarm signals to clinicians and educators in the modern world.

## Author Contributions

All authors listed have made a substantial, direct and intellectual contribution to the work, and approved it for publication.

## Conflict of Interest

The authors declare that the research was conducted in the absence of any commercial or financial relationships that could be construed as a potential conflict of interest.
