# Supplementary material for: Excessive Smartphone Use Is Associated With Health Problems in Adolescents and Young Adults
Source: Front Psychiatry. 2021 May 28;12:669042. doi: 10.3389/fpsyt.2021.669042 (PMC8204720; doi:10.3389/fpsyt.2021.669042)
Supplement: Supplementary file 1 [file Table_1.DOCX]

| ***Citation*** | ***Population*** | ***Main findings*** | |  |
| --- | --- | --- | --- | --- |
| Lee et al., (2017) [4] | 370 middle school students in South Korean | Being female, preoccupation, conflict, and use for ubiquitous trait predicted excessive smartphone use. | | **Predictors of excessive smartphone use** |
| Lee et al., (2018) [5] | 1029 South Korean adolescents | Excessive use of smartphones was correlated with impairment in the function of the family and relationship with friends, impulsiveness, and low self-esteem. | |  |
| Liu et al., (2016) [6] | 880 adolescents in Taiwan | Smartphone gaming was associated with excessive smartphone use. | |  |
|  |  |  | | **Sensation seeking and boredom** |
| Turgman et al., (2020) [7] | 140 university students in Israel | The interaction between high sensation seeking and abstinence whereby abstinence for 1.5 hours increased excessive smartphone use ratings in high sensation seeking students. | |  |
| Jiang et al., (2018) [8]  Leung., (2007) [9]  Lepp., (2014) [10]  Roberts., (2014) [11]  Yildiz., (2019 [12] | 616 University students in North China.  624 teenagers and young adults (age from 14 to 28) in Hong Kong.  536 Undergraduate students in the USA.  164 College undergraduates in the USA.  612 secondary and high school students in Turkey. | Boredom, avoidance of uncomfortable situations and the need for entertainment predicted excessive smartphone use. | |  |
| Lepp et al., (2019) [13] | 228 undergraduate students in the USA | Predictor of excessive sedentary behavior (e.g. watching videos) | |  |
| Ben-Yehuda et al., (2016) [14] | 40 University students in Israel. | Excessive smartphone use was not influenced by any interest or involvement in daily activities, indicating a compulsive behavior. | |  |
| Li et al., (2015) [15] | 516 Undergraduate college students | Poor sleep quality, reduced academic performance, and reduced subjective well-being among individuals with an external locus of control. | |  |
|  |  |  | | **Insecure attachment, poor cognitive-emotional regulation and communication problems** |
| Ali et al., (2020) [16]  Eichenberg et al., (2019) [17] | 114 mothers with infants in Jordan.  497 participants  (age 17–70, M= 19.38) | Insecure attachment positively correlated with problematic smartphone use. | |  |
| Extremera et al., (2019) [18] | 845 Spanish adolescents | Problematic smartphone users reported high scores in maladaptive cognitive-emotion regulation. | |  |
| Ruiz-Ruano et al., (2020) [19] | 1176 participants (age 16-82) | Experiential avoidance has been associated with excessive smartphone use and social networks. | |  |
| Emirtekin et al., (2019) [20]  Domoff et al., (2020) [21]  Mahapatra (2019) [22] | 443 high-school students in  Turkey.  193 adolescents  350 students (age 15-20) | Childhood emotional maltreatment, emotion regulation difficulties, unregulated eating, restrained eating, food addiction, higher percent body fat, and loneliness were associated with excessive smartphone use. | |  |
| Elhai et al., (2019) [23] | 302 American undergraduate students | Worry and anger are correlated with problematic smartphone use. | |  |
| Elhai et al., (2020) [24] | 316 American undergraduate students. | Excessive reassurance seeking behavior mediated the association between rumination and problematic smartphone use. | |  |
| Hashmi et al., (2019) [25]  Celikkalp et el., (2020) [26] | 700 Medical students in Pakistan.  502 Nursing students at a public university. | Poor communication skills were correlated with excessive smartphone use. | |  |
| Kim (2019) [27] | 288 participants (age 13-40) | Excessive use of the smartphone has been associated with reduce face-to-face interactions, and increase loneliness. | |  |
|  |  |  | | **Impaired cognitive function** |
| Chen et al., (2016) [28] | 38 University students | Problems in inhibitory control mechanisms in excessive smartphone users. | |  |
| Hadar et al., (2017) [29] | 46 University students | Impaired attention, reduced numerical processing capacity, increased impulsivity, hyperactivity and negative social concern in heavy smartphone users. | |  |
| Wegmann et al., (2020) [30] | 112 participants (age 17-53) | Increased problematic social networks use is associated with higher impulsivity, especially if executive functions or specific inhibitory control were impaired. | |  |
|  |  |  | | **Social media use, personality and impulsivity** |
| Gugushvili et al., (2020) [31]  Wolniewicz et al., (2018) [32]  Eide et al., (2018) [33] | 426 Estonian participants (age 18-56)  299 college students    127 participants (age 18-48) | Problematic social media use has been shown to be associated with "fear of missing out" (FOMO). | |  |
| Sha et al., (2019) [34] | 2299 participants | FOMO mediated the relationships between anxiety and depression with problematic smartphone use. | |  |
| He et al., (2020) [35] | 668 college students | Excessive smartphone use has been associated with social comparisons on social networking sites and perceived stress. | |  |
| Herrero et al., (2019) [36]  Hussain et al., (2017) [37]  Mitchell et al., (2018) [38]  Pivetta et al., (2019) [39] | 526 smartphone users in Spain  640 smartphone users (13-69)  153 smartphone users (age 18-68)  511 smartphone users (age 13-68) | Personality factors such as conscientiousness, openness, emotional stability, neuroticism, impulsivity, and excessive reassurance seeking have been associated with problematic smartphone use. | |  |
|  |  |  | | **Comorbidity with anxiety, depression OCD, ADHD and Alcohol use disorder** |
| Turgman et al., (2020). [7]  Yildiz Durak (2017) [12]  Demirci et al., (2015) [40]  Kim et al., (2020) [43]  Tangmunkongvorakul et al., (2019) [48] | 140 University students  612 secondary and high school students  319 University students  54,603 South Korean adolescents (26,930 males and 27,673 females)  800 University students | Excessive smartphone use has been associated with depression, anxiety and social anxiety. | |  |
| Enez Darcin et al., (2016) [44] | 367 University students | Social anxiety and loneliness | |  |
| Elhai et al., (2018) [41]  Elhai et al., (2018) [42]  Matar Boumosleh and Jaalouk (2017) [45]  Kim et al., (2018) [47]  Bhatt et al., (2019) [49]  Elhai et al., (2020) [50]  Coyne et al., (2019) [52]  Jeong et al., (2020) [54]  Squires et al.,(2020) [57] | 261 college students  296 college students  688 University students  4854 adults (age 19–49)  320 dental students in India  295 college students  385 participants (age 17-19)  714 middle school students in South Korea  204 undergraduate students | Depression and anxiety | |  |
| Kim et al., (2020) [46] | 189 participants with internet gaming disorder | Low self-esteem | |  |
| Elhai et al., (2020) [51] | 908 residents of a large Eastern Chinese city (age 17-64) | Depression, anxiety and COVID-19 anxiety | |  |
| Ivanova (2020) [53] | 402 university and college students from Ukraine, aged (17 – 31) | Depression | |  |
| Jinhee et al., (2020) [55] | 62,276 Middle- and high-school students | Depressive mood and suicidal ideation | |  |
| Karsay et al., (2019) [56] | 461 participants (18- 65) | Stress and loneliness | |  |
| Kempf et al., (2020) [58] | 633 adults (age 18–65) | OCD symptoms in those who used their smartphone for 2 or more hours per day | |  |
| Kim et al., (2019) [59] | 4512 Middle- and high-school students | ADHD predicted excessive smartphone use | |  |
| Beison and Rademacher (2017) [60] | 100 University students | Family history of alcoholism and father's education level explained 26% of the variance of problematic smartphone use | |  |
| Grant et al., (2019) [61] | 31,425 University students | Alcohol use disorder symptoms, impulsivity, PTSD, anxiety, depression and enhanced social activity. | |  |
| Contractor et al., (2017) [62] | 346 participants | Impulsivity mediated the relationship between PTSD severity and problematic smartphone use. | |  |
|  |  |  | | **Medical complications- sleep, physical fitness, eyesight, migraine and pain** |
| Kim et al., (2020) [63] | 350 children (aged 5-8) in South Korea | Reduced sleep time and reductions in sleep quality | | Sleep disturbances |
| Lemola et al., (2015) [64] | 362 adolescents | Sleep disturbance partially mediated the relationship between electronic media use in bed before sleep and symptoms of depression | |  |
| Dewi et al., (2018) [65] | 714 Adolescents | Sleep disturbance and depression symptoms | |  |
| Ghekiere et al., (2019) [66] | 671,083 Adolescents in a major study of 33 European and a non-European countries | Increase in the prevalence of sleep-onset difficulties and a small increase in physical activity. Adolescents exceeding 2-hours daily screen time had 20% higher odds of reporting sleep-onset difficulties, while no association was found for physical activity. | |  |
| Twenge **et al., (2019)** [67] | A 2016 national US survey of caregivers (n=43,755) children and adolescents (ages 0-17) | Fewer hours of sleep and insufficient sleep. Portable electronic devices have a stronger association with sleep duration than non-portable electronic screens in children over age 10. | |  |
| Tamura et al., (2017) [68] | 295 high school students aged 15–19 in Japan | Shorter sleep duration and insomnia. | |  |
| Liu et al., (2019) [69] | 4733 students (aged 14-24) in a technical college in China | Sleep disturbances, depression, and anxiety. | |  |
| Akhtar et al., (2019) [70] | 500 University students in Pakistan | Disturbed sleep pattern and poor sleep quality | |  |
| Dharmadhikari et al., (2019) [71] | 195 Medical students in India | Poorer sleep quality and higher perceived stress. | |  |
| Huang et al., (2020) [72] | 439 Chinese college students | Poor sleep quality | |  |
| Kim et al., (2015) [73] | 110 Chinese students in South Korea | Less physical activity and body composition, such as muscle mass and fat mass. | |  |
| Lee at el., (2016) [74] | 12 patients with acute acquired comitant esotropia (AACE) | Excessive smartphone use might influence acquired comitant esotropia (AACE) (an inward turning of the eye) development in adolescents. | | Problems in eyesight |
| Kim et al., (2016) [75] | 715 Adolescents | Higher prevalence rates for ocular symptoms. | |  |
| Demirci et al., (2016) [76] | 242 Turkish university students^54^ | Headache complaints, Duration and frequency of headache attack, were significantly higher in high smartphone users compared with low smartphone users | | Headaches and migraines |
| Montagni et al., (2016) [77] | 4927 French students | Students with the highest screen time exposure had an increased risk for migraine. | |  |
| Demir et al., (2019) [78] | 123 Migraine patients | Smartphone use has increased headache duration and frequency in. Its overuse was related to poor sleep quality and daytime sleepiness; and decreased quality of life. | |  |
| Zhuang et al., (2020) [79] | 2438 Young chronic neck pain patients | Patients with overuse of smartphones had higher Cervical Disc Degeneration Scale (CDDS) scores | | Chronic pain |
| İnal et al., (2015) [80] | 102 Young students | Higher median nerve Cross sectional areas (CSA's) in their dominant hands. Measures of excessive smartphone use correlated with subjective measures of pain for movement and rest and pinch strength. Smartphone overuse enlarges the median nerve, causes pain in the thumb, and decreases pinch strength and hand functions | |  |
| ***Citation*** | ***Population*** | ***Main findings*** | ***Methods*** |  |
| Tymofiyeva et al., (2020) [81] | 19 adolescents 11 males and 8 females) | Excessive smartphone use has been correlated with right amygdala activity. Excessive smartphone use has also been associated with sleep problems and depressive symptoms, particularly in females | Magnetic Resonance Imaging (fMRI) | **Brain imaging** |
| **Chun et al., (2017)** [82] | Twenty-five excessive smartphone users and 27 control partcipants. | A study assessing facial emotional processing and the Behavioral Inhibition System/Behavioral Activation System (BIS/BAS) in fMRI has shown neural deactivation in the DLPFC and dorsal ACC during the presentation of an angry face and emotional transition compared to control participants. The excessive smartphone use group showed neural deactivation of the STS and temporal-parietal junction related to social interaction during emotional transition compared to control participants.  The BAS-Reward Responsiveness level correlated with behavioral responses during repeated happy faces related to emotional reward in the excessive smartphone use group compared to control participants. | Functional Magnetic Resonance Imaging (fMRI) |  |
| Chun et al., 2018 [83] | Thirty-eight adolescents with excessive smartphone use and forty-two healthy control participants (HC). | Excessive smartphone users showed lower functional connectivity between the right OFC and the NAcc, and between the left OFC and MCC. Functional connectivity between the MCC and NAcc was greater in excessive smartphone users. Severe withdrawal symptoms were associated with higher cortisol concentrations and negatively correlated with OFC connectivity with the NAcc. | Functional Magnetic Resonance Imaging (fMRI) |  |
| **Lee et al., (2019) [84]** | Thirty-nine problematic smartphone users with excessive use of social networking platforms via smartphone and 49 normal control male and female smartphone users. | Problematic smartphone users had smaller Frontal-Cingulate Gray Matter Volume (GMV) in the right OFC.  Negative correlations between GMV in the right lateral OFC and the Smartphone Addiction Proneness Scale (SAPS) score, including the SAPS tolerance subscale in excessive users, implying that lateral OFC gray matter abnormalities are implicated in problematic smartphone use, especially in social networking overuse. Individuals with excessive smartphone use showed lower GMV in left anterior Insula, inferior temporal and parahippocampal gyrus | Gray matter volume measure in MRI |  |
| Horvath et al., (2020) [85] | Twenty-two participants with smartphone addiction and twenty six control participants. | Lower activity in the right ACC in individuals with excessive smartphone use.  A negative correlation between individuals with excessive smartphone use and both ACC GMV and activity.  A negative association between individuals with excessive smartphone use scores and left OFC GMV. | Gray matter volume and brain activation in fMRI |  |
| Paik et al., (2019) [86] | Ninety smartphone users (33 males and 57 females) | The strength of the resting state functional connectivity (rsFC) between the left Insula and right putamen, and between the right insula and left superior frontal, middle temporal, fusiform, inferior OFC and right STG in fMRI positively correlated with smartphone time in bed. | Resting state functional connectivity (rsFC) in fMRI |  |
| Schmitgen **et al., (2020) [87]** | Twenty-one participants with smartphone addiction and twenty one control participants. | Exposure to smartphone pictures in fMRI was associated with activation of the frontal operculum/anterior Insula and precentral gyrus in active smartphone users.  Negative correlations between MPC, ACC, precuneus, and precentral gyrus and specific smartphone addiction scores such as compulsive behavior, functional impairment and withdrawal. | Brain activation in fMRI |  |

^1^ The studies are arranged in order of the appearance in the article.

^2^ Abbreviations: dorsolateral prefrontal cortex (DLPFC), Orbito-Frontal Cortex (OFC), Anterior Cingulate Cortex (ACC) Nucleus Accumbens (NAcc), Mid Cingulate Cortex (MCC) .Superior Temporal Sulcus (STS).
